# Effects of *OPRM1* and *ABCB1* gene polymorphisms on the analgesic effect and dose of sufentanil after thoracoscopic-assisted radical resection of lung cancer

**DOI:** 10.1042/BSR20181211

**Published:** 2019-01-03

**Authors:** Zhonghai Zhao, Bin Lv, Xiaodong Zhao, Yunlong Zhang

**Affiliations:** 1 Department of Cardiothoracic Surgery, Yidu Central Hospital of Weifang, Qingzhou, China; 2 Department of Anesthesiology, Yidu Central Hospital of Weifang, Qingzhou, China; 3 Department of Anesthesiology, Zhejiang Provincial People’s Hospital, People’s Hospital of Hangzhou Medical College, Hangzhou, China

**Keywords:** ABCB1 gene, Radical cancer, Sufentanil, Single nucleotide polymorphism, μ-opioid receptor gene

## Abstract

Objective: To study the effects of single-nucleotide polymorphisms of the *OPRM1* and *ABCB1* genes on the analgesic effect and consumption of sufentanil after thoracoscopic-assisted radical resection of lung cancer.

Methods: A total of 225 Chinese Han nationality patients undergoing thoracoscopic-assisted radical resection of lung cancer were enrolled in the present study. Among them, 132 were males (58.67%) and 93 (41.33%) were females having American Society of Anesthesiologists statuses classified as grades I or II. The rs1799971, rs563649 and rs1323040 genotypes of the *OPRM1* gene and rs2032582, rs1045642 and rs1128503 genotypes of the *ABCB1* gene were detected by Sanger sequencing. The state anxiety index and pressure pain threshold were assessed preoperatively. Sufentanil was administered intravenously to maintain anesthesia. The doses and side effects of sufentanil consumed 6 h (T1), 24 h (T2) and 48 h (T3) after surgery were recorded.

Results: The sufentanil doses at T1, T2 and T3 were significantly higher in radical-operation lung cancer patients with mutant homozygous rs1799971 and rs1323040 loci in the *OPRM1* gene and rs2032582 and rs1128503 loci in the *ABCB1* gene. The doses of sufentanil consumed by mutant heterozygous lung cancer patients at T1, T2 and T3 were significantly higher than those consumed by patients without mutations, and the differences were statistically significant (*P*<0.05). There was no significant difference in sufentanil doses consumed by lung cancer patients with mutant homozygous, mutant heterozygous and wild-type rs563649 locus of the *OPRM1* gene and rs1045642 locus of the *ABCB1* gene at T1, T2 and T3 (*P*>0.05). There was no significant difference in the visual analog scale scores at T1, T2 and T3 for different genotypes of *OPRM1* and ABCB1 genes in lung cancer patients (*P*>0.05). No significant difference was found between the adverse reactions of *OPRM1* and ABCB1 genotypes in patients undergoing radical resection of lung cancer (*P*>0.05).

Conclusion: The rs1799971 and rs1323040 polymorphisms of the *OPRM1* gene and rs2032582 and rs1128503 polymorphisms of the *ABCB1* gene are related to the analgesic effect and consumed dose of sufentanil in Chinese Han patients undergoing radical operation of lung cancer.

## Introduction

Lung cancer is one of the most common malignant tumors in the world with high mortality and morbidity as well as the propensity to metastasize. Currently, radical surgery combined with chemoradiotherapy is the primary means of lung cancer treatment [1,2]. However, the perception of pain and response to analgesics vary among patients treated with radical cancer surgery because of age, physical condition and severity of disease. Genetic polymorphism is also one of the most important factors for such individual differences [3,4].

The μ-opioid receptor, encoded by the human opioid receptor μ-1 gene (*OPRM1*), is the primary site of action for sufentanil. Recent studies found that single-nucleotide polymorphisms (SNPs) of *OPRM1* significantly influence the analgesic effectiveness and side effects of sufentanil [5–7]. Sufentanil is one of the inhibitors of P-glycoprotein (P-gp) [8], which belongs to the superfamily of ATP-binding cassette (ABC) transporters and is the most important efflux transporter of exogenous opioids [9]. P-gp in humans is encoded by the gene of ATP-binding cassette sub-family B member 1 (*ABCB1*), with at least 38 SNPs. Several studies reported that *ABCB1* genetic polymorphisms were correlated with the analgesic effectiveness and consumption of opioids [10].

This work aims to analyze the effects of SNPs of the *OPRM1* gene, including rs1799971 A>G (*Asp40Asn*), rs563649 C>T, rs1323040 A>G and SNPs of the *ABCB1* gene, such as rs2032582 G>T, rs1045642 C>T, rs1128503 C>T, on the analgesic effect and consumption of sufentanil in patients treated with thoracoscopic-assisted radical resection of lung cancer. The polymorphisms of *OPRM1* rs1799971 and rs1323040 loci and of *ABCB1* rs2032582 and rs1128503 loci have been recently brought into research focus. The mutation of the *OPRM1* rs1799971 locus resulted in the amino acid change from Asn to Asp, which was related to the endogenous stress reaction intensity and could decrease the susceptibility to heroin recurrence in patients without opioid receptor agonist [11]. In addition, the rs563649 C>T and rs1323040 A>G mutations were reported to influence mRNA levels and translation efficiencies [12]. Studies have shown that the rs1128503 C>T mutation in *ABCB1* gene is a risk factor in paclitaxel-induced peripheral neuropathy, while the rs2032582 G>T and rs1045642 C>T mutations have no significant correlation with paclitaxel-induced peripheral neuropathy [13].

## Materials and methods

### Participants

Approved by the hospitals’ Ethics Committees (No. 201404312L), a total of 225 patients from Yidu Central Hospital of Weifang and Tongde Hospital of Zhejiang Provinces who underwent thoracoscopic-assisted radical resection of lung cancer were recruited to the present study from May 2014 to July 2017. All enrolled patients were Han Chinese, with 132 men (58.67%) and 93 women (41.33%) aged 41–68 years, and had American Society of Anesthesiologists physical statuses I or II, which were evaluated by two senior anesthesiologists in our hospital before anesthesia. Patients with coronary artery disease, hypertension, diabetes and a history of liver–kidney dysfunction were excluded from the present study. Signed informed consent was obtained from all patients.

### Anesthetic analgesia

Before the initiation of anesthesia, we measured the baseline noninvasive blood pressure, electrocardiogram, oxygen saturation and end-tidal carbon dioxide partial pressure. Before surgery, the right internal jugular vein puncture and cannulation for CVP monitoring and transradial puncture for invasive blood pressure were carried out. For all patients, thoracic paravertebral block (TPVB) was performed under the guidance of ultrasound: the patients were in the lateral decubitus position, with arched back and bended knees, and conventional disinfection was performed. According to the operative incision, for the intercostal nerve block, the corresponding paravertebral space (T4-7) was selected, and the spinal Tuohy needle (18-gauge, 15 cm) was inserted 2.5 cm from the superior edge of the thoracic vertebra to withdraw the needle without bleeding. TPVB was accomplished by slowly injecting 15 ml of 0.5% ropivacaine. For anesthesia induction, an intravenous injection was done to provide the following doses: 0.05 mg/kg midazolam, 0.5 μg/kg sufentanil, 1–2 mg/kg propofol and 1 mg/kg rocuronium bromide. For anesthesia maintenance, drugs providing the doses of 6 to 9 mg/kg·h propofol, 6 to 10 μg/kg·h remifentanil and 0.05 mg/kg cis-atracurium were administered every 30–40 min to maintain muscle relaxation, using the bispectral index (BIS) monitor (Aspect, U.S.A.) to monitor the depth of sedation (BIS controlled between 45 and 60). After surgery, all patients were treated with patient-controlled epidural analgesia (PCEA). The regimen was set as follows: 2 μg/kg sufentanil diluted to 100 ml with saline, background infusion = 2 ml/h, demand dose = 1 ml, lockout interval = 20 min.

### Genotyping and single-nucleotide polymorphism

**Figure 1:**
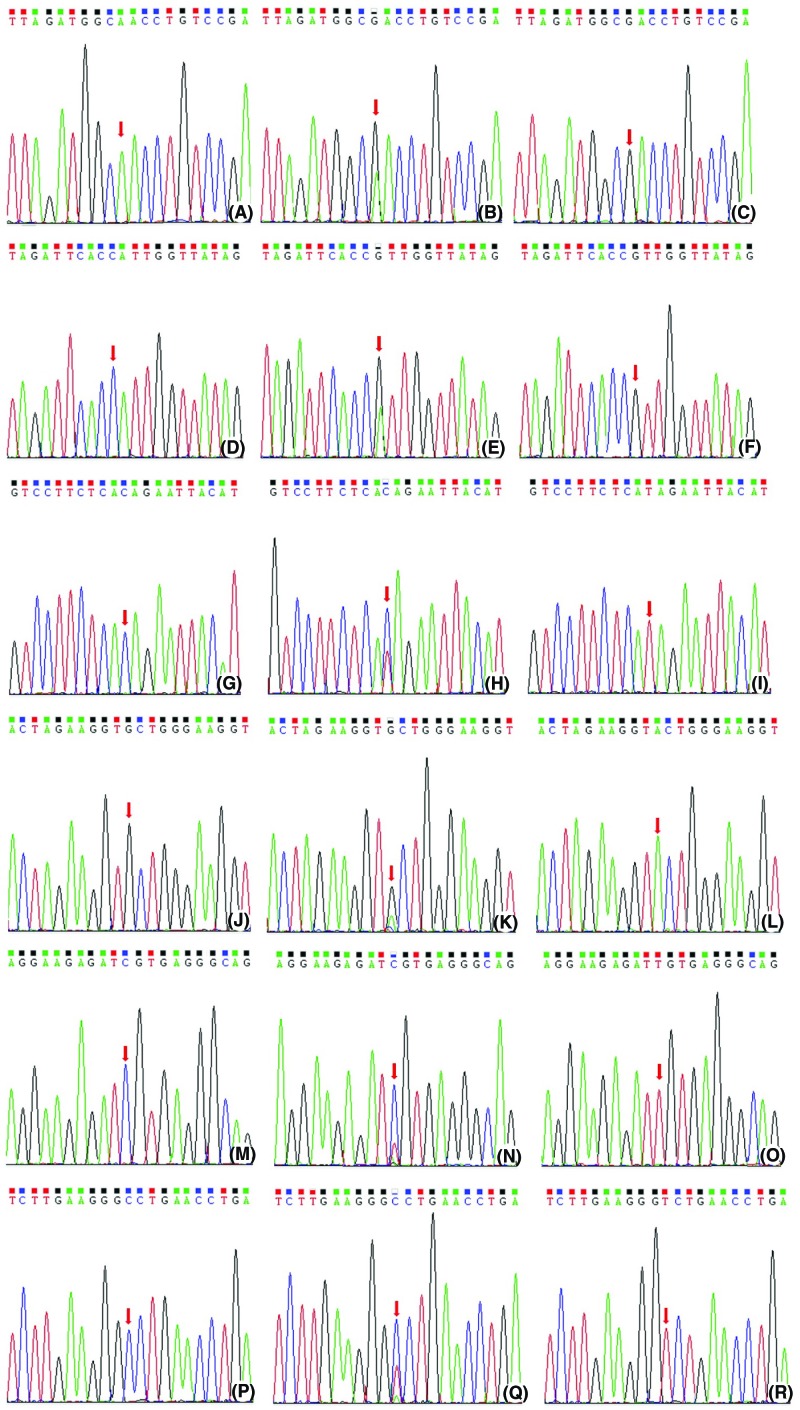
The Sanger sequence map of OPRM1 and ABCB1 gene polymorphisms (**A**) The AA genotype at rs1799971 locus in the OPRM1 gene. (**B**) The AG genotype at rs1799971 locus in the OPRM1 gene. (**C**) The GG genotype at rs1799971 locus in the OPRM1 gene. (**D**) The AA genotype at rs563649 locus in the OPRM1 gene. (**E**) The AG genotype at rs563649 locus in the OPRM1 gene. (**F**) The GG genotype at rs563649 locus in the OPRM1 gene. (**G**) The CC genotype at rs1323040 locus in the OPRM1 gene. (**H**) The CT genotype at rs1323040 locus in the OPRM1 gene. (**I**) The TT genotype at rs1323040 locus in the OPRM1 gene. (**J**) The GG genotype at rs2032582 locus in the ABCB1 gene. (**K**) The GT genotype at rs2032582 locus in the ABCB1 gene. (**L**) The TT genotype at rs2032582 locus in the ABCB1 gene. (**M**) The CC genotype at rs1045642 locus in the ABCB1 gene. (**N**) The CT genotype at rs1045642 locus in the ABCB1 gene. (**O**) The TT genotype at rs1045642 locus in the ABCB1 gene. (**P**) The CC genotype at rs1128503 locus in the ABCB1 gene. (**Q**) The CT genotype at rs1128503 locus in the ABCB1 gene. (**R**) The TT genotype at rs1128503 locus in the ABCB1 gene.

Venous blood samples (2 ml) were collected from all patients, and genomic DNA was isolated from white blood cells using a Qiagen Genomic DNA Isolation Kit (51104, Qiagen, Valencia, CA, U.S.A.). The *OPRM1* and ABCB1 genes were amplified by polymerase chain reaction (PCR) using the forward and reverse primers shown in Table 1, with PCR reaction conditions as follows: 25 mM MgCl_2_, 1 mM dNTPs, 100 pmol/μl forward and reverse primers, 1U Taq polymerase and 10 ng DNA. After PCR, the purified PCR products were analyzed with the Sanger sequencing method to identify the genotypes of *OPRM1* and *ABCB1* SNPs, shown in Figure 1.

**Table 1:** The primer sequences for OPRM1 and ABCB1 gene amplification

Gene locus	SNPs	Primer	PCR reaction condition
OPRM1	rs1799971	Forward: 5′-TGATGAGCCTCTGTGACTGC-3′;	35 cycles: 95°C, 30 s
		Reverse: 5′-CTTAAGCCGCTGAACCCT-3′	58°C, 45 s, 72°C, 45 s
	rs563649	Forward: 5′-GCTGGGTAGGAAAGTGGCAA-3′;	35 cycles: 95°C, 30 s
		Reverse: 5′-TGACCTTGGTGCTCAAGAAGT-3′	58°C, 45 s, 72°C, 45 s
	rs1323040	Forward: 5′-GCTGGGTAGGAAAGTGGCAA-3′;	35 cycles: 95°C, 30 s
		Reverse: 5′-TGACCTTGGTGCTCAAGAAGT-3′	60°C, 45 s, 72°C, 45 s
ABCB1	rs2032582	Forward: 5′-TCAGCATTCTGAAGTCATGGAA-3′;	35 cycles: 95°C, 30 s
		Reverse: 5′-TTAGAGCATAGTAAGCAGTAGGGAGT-3′	60°C, 45 s, 72°C, 45 s
	rs1045642	Forward: 5′-GTGTGCTGGTCCTGAAGTTG-3′;	35 cycles: 95°C, 30 s
		Reverse: 5′-TGGAGCCTCAAGCCTATAGC-3′	58°C, 45 s, 72°C, 45 s
	rs1128503	Forward: 5′-GTTCACTTCAGTTACCCATCTCG-3′;Reverse: 5′-CGTGGTGGCAAACAATACAGG-3′	35 cycles: 95°C, 30 s, 60°C, 45 s, 72°C, 45 s

### Data collection

Basic information including age, sex, height and weight of the patients was recoded. The pressure pain threshold (PPT) was evaluated 15 min before surgery using a calibrated Touch-Test Sensory Evaluator (North Coast Medical, Gilroy, CA, U.S.A.) by the 0.1-cm^2^ probes in patients’ right forearm. We recorded the consumption of sufentanil with a PCEA pump during the first-operative 6 h (T1), 24 h (T2) and 48 h (T3). The pain score during surgery was evaluated on a visual analog scale (VAS): point 0 meant painlessness, while the point 10 meant intense pain. When the VAS score of the patient treated with PCEA was maintained at 3 or lower, patients’ satisfaction evaluation for analgesic effect at T3, and the side effects (including nausea, vomiting, respiratory depression and pruritus) were recorded.

### Statistical analysis

All analyses were performed with SPSS 20.0 (SPSS Inc., Chicago, IL, U.S.A.). The genotype distribution was tested for the Hardy–Weinberg equilibrium with the χ^2^ test. One-way ANOVA was used in the analysis of the differences in demographic data, sufentanil consumption and VAS scores among the three genotype groups. The difference between two genotype groups was evaluated with the Mann–Whitney *U*-test and the Student’s *t*-test. After single-factor analysis of variance, the Kruskal–Wallis and Friedman tests, the Bonferroni multiple comparison test was used for correction. The χ^2^ test and the Fisher’s exact test were used for analysis of the differences in side-effect incidence rates among three genotype groups. A two-tailed *P*-value less than 0.05 was considered to indicate statistical significance.

## Results

### Genotype distributions of *OPRM1* and *ABCB1* SNPs

Genotype distributions of *OPRM1* SNPS (rs1799971, rs563649 and rs1323040) and ABCB1 SNPs (rs2032582, rs1045642 and rs1128503) are shown in Table 2, and the Sanger sequencing results are shown in Figure 1. The genotype frequencies of these six SNPs of two genes are in agreement with the Hardy–Weinberg equilibrium (*P*>0.05). The mutation of A allele to G allele was detected at rs1799971 and rs563649 genetic loci of the *OPRM1* gene, and the mutation of C allele to T allele was detected at rs20832582 genetic locus of the *OPRM1* gene. No other mutations were detected. The mutation of G allele to A allele was detected at rs2032582 genetic locus of the ABCB1 gene, and the mutation of C allele to T allele was detected at rs1045642 and rs1128503 genetic loci of the ABCB1 gene. In addition, general information including age, sex ratio, BMI, ASA score, PPT and SAI for patients with wild-type homozygotes, mutant-type heterozygotes and mutant-type homozygotes of different SNPs showed no significant differences (*P*>0.05), as shown in Table 2.

**Table 2:** The general information of OPRM1 and ABCB1 SNPs in 225 patients underwent radical section of lung cancer

SNPs	Genotype	Patients (*n*)	Age (years)	Sex (men/women)	BMI (kg/m^2^)	ASA score (I/II)	PPT (g)	SAI (low/high)
OPRM1 rs1799971	AA	115	52.35 ± 10.55	68/42	22.43 ± 3.16	61/41	96.85 ± 52.01	95/20
	AG	78	51.98 ± 11.24	44/34	22.98 ± 2.94	42/31	99.75 ± 49.87	71/7
	GG	32	53.30 ± 12.45	20/17	23.04 ± 3.12	29/21	94.86 ± 50.61	29/3
OPRM1 rs563649	AA	104	53.15 ± 11.76	61/43	22.86 ± 2.85	58/39	92.94 ± 57.43	85/19
	AG	78	51.43 ± 12.34	43/35	23.14 ± 3.02	43/36	98.54 ± 49.85	65/13
	GG	43	52.43 ± 12.04	28/15	22.54 ± 2.46	31/18	96.87 ± 52.36	36/7
OPRM1 rs1323040	CC	124	51.45 ± 13.01	71/50	23.14 ± 2.75	67/48	93.45 ± 53.24	103/21
	CT	79	52.51 ± 12.34	42/34	22.86 ± 3.10	41/31	97.52 ± 54.25	67/12
	TT	22	53.09 ± 12.14	19/9	23.05 ± 3.14	24/14	105.24 ± 58.98	16/6
ABCB1 rs2032582	GG	109	53.06 ± 11.85	65/44	22.65 ± 3.24	61/40	95.64 ± 49.68	91/18
	GA	85	52.49 ± 12.42	49/36	23.10 ± 3.16	47/35	96.54 ± 52.14	74/11
	AA	31	52.41 ± 11.97	18/13	22.56 ± 2.98	24/18	94.38 ± 53.64	24/7
ABCB1 rs1045642	CC	120	53.08 ± 12.45	69/48	23.08 ± 3.24	68/49	96.52 ± 41.65	97/23
	CT	58	52.14 ± 11.35	35/26	22.64 ± 2.98	37/25	94.52 ± 46.54	47/11
	TT	47	51.78 ± 12.04	28/19	22.89 ± 3.06	27/19	96.54 ± 50.24	38/9
ABCB1 rs1128503	CC	131	52.45 ± 11.38	74/56	23.14 ± 3.24	72/54	95.84 ± 48.75	105/26
	CT	63	53.64 ± 12.34	39/25	22.99 ± 2.98	40/26	97.84 ± 53.21	50/13
	TT	31	51.79 ± 13.06	19/12	23.07 ± 3.24	20/13	101.52 ± 53.24	27/4

Note: *P<0.05, compared with wide-type homozygotes; ASA, American Society of Anesthesiologists; BMI, body mass index, PPT, pressure pain threshold, SAI, state anxiety index.

### Genotypes of *OPRM1* and *ABCB1* SNPs with sufentanil consumption and VAS scores

The total consumption of PCEA sufentanil and the VAS scores for different genotypic groups are shown in Table 3. Among patients who underwent radical resection of lung cancer, the consumption of PCEA sufentanil at T1, T2 and T3 in the patient groups carrying mutant-type homozygotes at rs1799971 and rs1323040 loci of *OPRM1* as well as rs2032582 and rs1128503 loci of ABCB1 was significantly increased compared with that of the wild-type homozygote and mutant-type heterozygote groups. The consumption of PCEA sufentanil at T1, T2 and T3 in the mutant-type heterozygote groups was significantly increased compared with that of wild-type patients. All differences had statistical significance (*P*<0.05). For the rs563649 locus of the *OPRM1* gene and the rs1045642 locus of the ABCB1 gene, there was no significant difference in the consumption of PCEA sufentanil at T1, T2 and T3 between mutant-type heterozygote, mutant-type homozygote and wild-type patients (*P*>0.05). There was no significant difference in the VAS scores at T1, T2 and T3 among different genotypes of *OPRM1* and ABCB1 SNPs (*P*>0.05).

**Table 3:** The consumption of PCEA sufentanil and the VSA scores among different genotypes

SNPs	Genotype	Patients (*n*)	VAS Score (point)			Sufentanil consumption (μg)		
			T1	T2	T3	T1	T2	T3
OPRM1 rs1799971	AA	115	2.32 ± 1.17	1.42 ± 1.15	1.36 ± 1.05	18.52 ± 3.14	64.35 ± 5.12	124.54 ± 8.15
	AG	78	2.47 ± 1.17	1.54 ± 1.13	1.39 ± 1.06	20.98 ± 2.77	67.59 ± 4.89	135.54 ± 7.81
	GG	32	2.56 ± 1.14	1.59 ± 1.17	1.42 ± 1.01	23.45 ± 2.67	71.52 ± 5.03	149.55 ± 8.14
OPRM1 rs563649	AA	104	2.46 ± 1.17	1.71 ± 1.15	1.51 ±1.09	19.54 ± 3.06	66.85 ± 4.23	128.78 ± 7.56
	AG	78	2.51 ± 1.12	1.63 ± 1.14	1.44 ± 1.04	20.04 ± 3.56	68.52 ± 4.85	131.46 ± 8.14
	GG	43	2.63 ± 1.16	1.81 ± 1.17	1.47 ± 1.07	20.43 ± 2.98	69.87 ± 3.89	155.55 ± 8.49
OPRM1 rs1323040	CC	124	2.51 ± 1.07	1.73 ± 1.13	1.38 ± 1.06	17.97 ± 3.24	63.54 ± 4.87	122.55 ± 7.99
	CT	79	2.62 ± 1.12	1.76 ± 1.12	1.44 ± 1.03	21.54 ± 3.14	67.71 ± 4.19	139.54 ± 8.46
	TT	22	2.69 ± 1.11	1.69 ± 1.14	1.51 ± 1.02	25.04 ± 3.61	75.24 ± 3.98	156.41 ± 7.43
ABCB1 rs2032582	GG	109	2.44 ± 1.07	1.47 ± 1.14	1.32 ± 1.05	18.17 ± 3.68	62.89 ± 4.25	125.85 ± 6.94
	GA	85	2.53 ± 1.07	1.52 ±1.15	1.44 ± 1.09	21.45 ± 3.15	68.79 ± 3.51	139.55 ± 7.12
	TA	31	2.62 ± 1.23	1.62 ± 1.12	1.48 ± 1.04	24.05 ± 3.16	73.15 ± 3.66	148.77 ± 7.46
ABCB1 rs1045642	CC	120	2.47 ± 1.09	1.74 ± 1.15	1.56 ± 1.02	19.54 ± 3.19	68.78 ± 5.04	131.66 ± 6.98
	CT	58	2.55 ± 1.16	1.64 ± 1.15	1.47 ± 1.09	20.42 ± 3.61	69.78 ± 4.29	133.98 ± 8.76
	TT	47	2.67 ± 1.04	1.59 ± 1.13	1.43 ±1.02	20.98 ± 3.65	71.05 ± 4.16	135.66 ± 8.49
ABCB1 rs1128503	CC	131	2.54 ± 1.12	1.71 ± 1.11	1.39 ± 1.03	17.96 ± 3.26	62.98 ± 5.68	123.64 ± 7.14
	CT	63	2.69 ± 1.06	1.54 ± 1.14	1.57 ± 1.02	20.79 ± 2.97	67.89 ± 4.26	138.96 ± 8.12
	TT	31	2.73 ± 1.14	1.63 ± 1.08	1.54 ± 1.08	23.45 ± 3.28	73.42 ± 3.97	147.88 ± 7.42

Abbreviation: VAS, visual analogue scale/score.

### Side effects

The side effects in patients who underwent radical resection of lung cancer under anesthetic analgesia included nausea (3.11%), vomiting (1.78%) and pruritus (0.89%), as shown in Table 4. There was no significant difference in the side effects among different genotypes of *OPRM1* and ABCB1 SNPs (*P*>0.05).

**Table 4:** Summary of the side effects of different genotype groups

SNPs	Genotype	Patients (*n*)	Nausea (%)	Vomiting (%)	Respiratory depression (%)	Pruritus (%)
OPRM1 rs1799971	AA	115	4 (3.48%)	3 (2.61%)	2 (1.74%)	1 (0.87%)
	AG	78	2 (2.56%)	1 (1.28%)	1 (1.28%)	1 (1.28%)
	GG	32	1 (3.13%)	0 (0)	0 (0)	0 (0)
OPRM1 rs563649	AA	104	3 (2.88%)	3 (2.88%)	2 (1.92%)	2 (1.92%)
	AG	78	2 (2.56%)	1 (1.28%)	0 (0)	0 (0)
	GG	43	2 (4.56%)	0 (0)	1 (2.33%)	0 (0)
OPRM1 rs1323040	CC	124	5 (4.03%)	4 (3.23%)	3 (2.42%)	2 (1.61%)
	CT	79	3 (3.80%)	0 (0)	0 (0)	0 (0)
	TT	22	0 (0)	0 (0)	0 (0)	0 (0)
ABCB1 rs2032582	GG	109	4 (3.67%)	4 (3.67%)	2 (1.8%)	1 (0.92%)
	GT	85	3 (3.53%)	0 (0)	1 (1.18%)	0 (0)
	TT	31	0 (0)	0 (0)	0 (0)	1 (3.23%)
ABCB1 rs1045642	CC	120	4 (3.33%)	3 (2.50%)	3 (2.50%)	2 (1.67%)
	CT	58	2 (3.45%)	0 (0)	0 (0)	0 (0)
	TT	47	1 (2.13%)	1 (2.13%)	0 (0)	0 (0)
ABCB1 rs1128503	CC	131	5 (3.82%)	4 (3.05%)	2 (1.53%)	1 (0.76%)
	CT	63	2 (3.17%)	0 (0)	1 (1.59%)	1 (1.59%)
	TT	31	0 (0)	0 (0)	0 (0)	0 (0)

## Discussion

Recently, genetic studies on narcotic analgesics have mainly focused on the correlation between gene polymorphisms and the consumption and analgesic effect of anesthetics. The present study focused on rs1799971, rs563649 and rs1323040 loci of the *OPRM1* gene and rs2032582, rs1045642 and rs1128503 loci of the ABCB1 gene, and we found that the SNPs at rs1799971 and rs1323040 loci of *OPRM1* and rs2032582 and rs1128503 loci of ABCB1 were significantly correlated with sufentanil consumption in patients who underwent radical resection of lung cancer. There was no significant difference in sex distribution of enrolled patients with respect to genotypes of different SNPs (*P*>0.05), which ruled out the sex difference effect on sufentanil consumption. Only intravenous infusions of sufentanil were administered for the maintenance of anesthesia, without any other anesthetic drug for all enrolled patients, and they received the same treatments by surgeons. Therefore, our results could point at the individual differences in the consumption of sufentanil for surgery and post-operative complications.

To date, most of the studies of the *OPRM1* gene have been focused on the rs1799971 gene locus, which is at the extracellular terminus 1. Mutation in rs1799971 leads to the Asp40Asn amino acid substitution [14]. Smith et al. [15] identified new loci upstream of OPRM1 through genome-wide association studies of therapeutic opioid drugs. Previous studies reported that the minor allele frequency (MAF, G allele) in 102 heroin addict patients who received methadone maintenance therapy was 0.449 [16], whereas in the present study, the MAF of enrolled patients was 0.316. The mutation at this gene locus could change amino acid sequence; therefore, we paid attention to the correlation between SNPs of this gene locus and analgesic effect and consumption of opioids. Our results showed that the SNPs of rs1799971 locus in *OPRM1* gene were significantly correlated with the consumption of PCEA sufentanil, rather than post-operative side effects in patients who underwent radical resection of lung cancer. However, the results of Liu et al. [3] showed that the SNP of rs558025 locus of *OPRM1* was correlated with the consumption of remifentanil during the gynecologic hysteroscopy surgery, while the SNP of the rs1799971 locus was unrelated. This contradiction might due to the sample size, duration of opioid use and ethnic genetic differences. In addition, we found that the sufentanil consumption in the patients carrying T allele at the rs1323040 locus in *OPRM1* had significantly increased, while SNPs of the rs563649 locus had no effect on the sufentanil consumption or on the post-operative side effects. This is the first study of the correlation between SNPs at the rs563649 and rs1323040 loci and the analgesic effect.

*The* ABCB1 (multidrug resistance gene1) gene polymorphisms have been associated with altered P-gp expression and activity, which impacted opioid metabolism [17–19]. Salvatore et al. [20] showed that Genome-wide association data suggest ABCB1 and immune-related gene sets may be involved in adult antisocial behavior. To date, there have been several clinical studies evaluating the correlation between ABCB1 gene polymorphisms with clinical data, including drug metabolism and risk of cancer [21–23]. The present study is the first to analyze the influence of SNPs at rs2032582, rs1045642 and rs1128503 loci in ABCB1 gene on the sufentanil consumption and analgesic effect in patients who underwent radical resection of lung cancer. Notably, we found that SNPs at the rs2032582 and rs1128503 loci in ABCB1 significantly affected sufentanil consumption, while SNPs at the rs1045642 locus had no effect. We thought that the mutation of G allele to A allele at rs2032582 and mutation of C allele to T allele at rs1128503 affected the protein function of P-gp, thereby increasing the consumption of sufentanil, which is the P-gp inhibitor [24–26]. Additionally, we evaluated the effects of SNPs of the *OPRM1* and ABCB1 genes on side effects and showed that SNPs had no significant effect on post-operative nausea, vomiting, respiratory inhibition and pruritus (*P*>0.05).

For the first time, our study evaluated the correlation between SNPs at the rs1799971, rs563649 and rs1323040 loci in *OPRM1* and the rs2032582, rs1045642 and rs1128503 loci in ABCB1 with sufentanil consumption in Chinese Han patients who underwent radical resection of lung cancer. However, due to the limited sample size, we have not enrolled other ethnic patients, who will be studied in the future. In addition, we have included SAI, PPT and ASA scores in the present study, which are also important and might affect the consumption and side effects of sufentanil. Our results showed that there was no significant difference among different genotypes in SAI, PPT and ASA scores, which ruled out their influence on the consumption and side effects of sufentanil.

## Conclusion

Our study is the first to evaluate the correlation between SNPs at the rs1799971, rs563649 and rs1323040 loci in the *OPRM1* gene and the rs2032582, rs1045642 and rs1128503 loci in the ABCB1 gene with sufentanil consumption in Chinese Han patients who underwent radical resection of lung cancer. The carriers of G allele at the rs1799971 locus and T allele at the rs1323040 locus in the *OPRM1* gene, as well as the carriers of A allele at the rs2032582 locus and T allele at the rs1128503 locus in the ABCB1 gene, consumed more sufentanil. Our results provide the evidence for the genetic factor effect on opioid pharmacokinetics. More studies are needed to define the functional sequence leading to the individual differences in opioids consumption and effect.

## Abbreviations

ABC: ATP-binding cassette
ABCB1: ATP-binding cassette sub-family B member 1
BIS: bispectral index
*OPRM1*: opioid receptor μ-1
PCEA: patient-controlled epidural analgesia
P-gp: P-glycoprotein
PPT: pressure pain threshold
SNP: single-nucleotide polymorphism
TPVB: thoracic paravertebral block
VAS: visual analog scale
